# Supplementary material for: Sudden Death of a Four-Day-Old Newborn Due to Mitochondrial Trifunctional Protein/Long-Chain 3-Hydroxyacyl-CoA Dehydrogenase Deficiencies and a Systematic Literature Review of Early Deaths of Neonates with Fatty Acid Oxidation Disorders
Source: Int J Neonatal Screen. 2025 Jan 26;11(1):9. doi: 10.3390/ijns11010009 (PMC11843868; doi:10.3390/ijns11010009)
Supplement: Supplementary file 1 [file IJNS-11-00009-s001.zip › IJNS-3326240-supplementary.pdf]

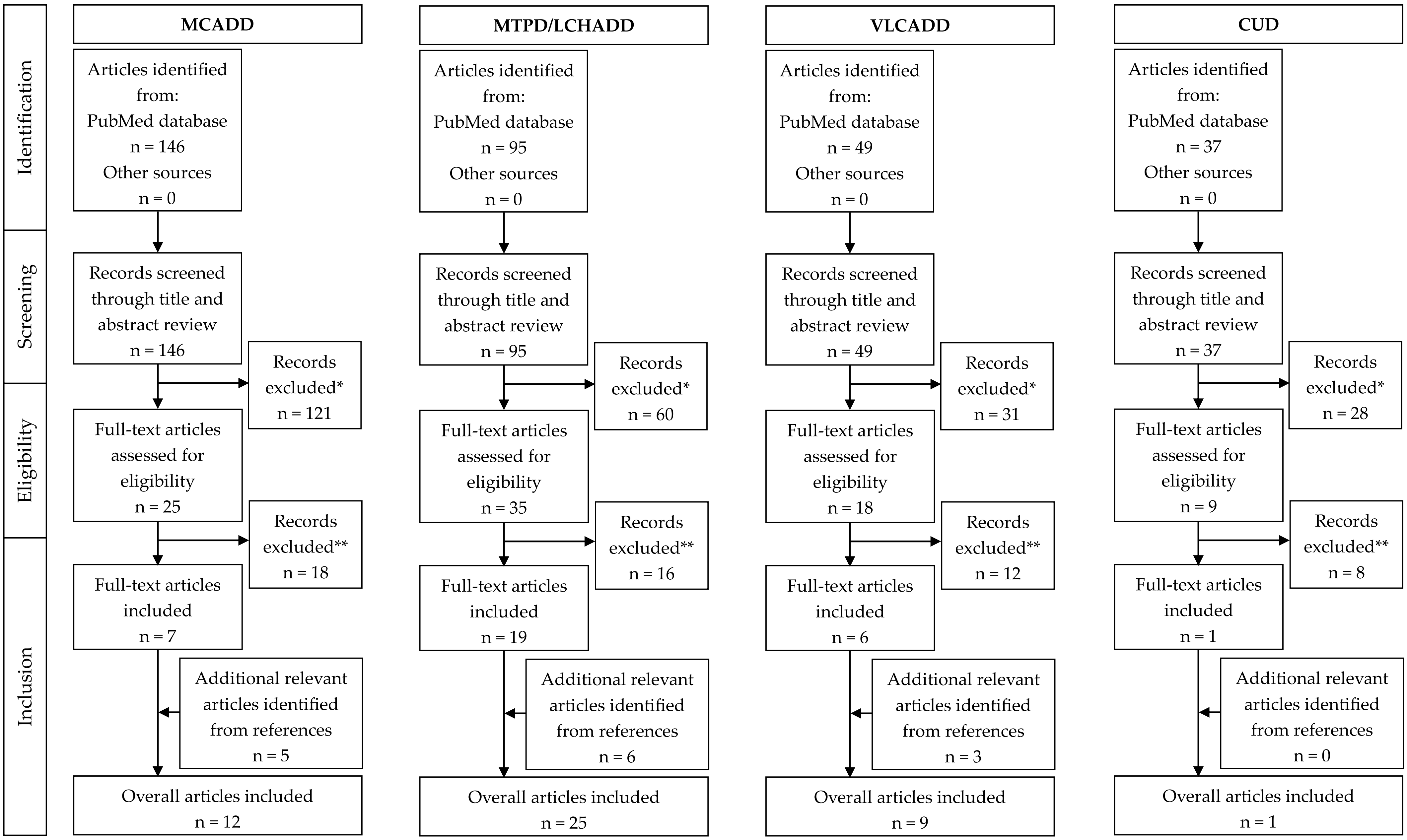


Figure S1: PRISMA flow diagram for a systematic literature review on early neonatal deaths due to FAODs. The diagram outlines a review of the following FAOD types: medium-chain acyl-CoA dehydrogenase deficiency (MCADD), mitochondrial trifunctional protein/long-chain 3-hydroxyacyl-CoA dehydrogenase deficiencies (MTPD/LCHADD), very long-chain acyl-CoA dehydrogenase deficiency (VLCADD), and carnitine uptake defect (CUD). Reasons for exclusion marked with *: no human data, articles not in English, records unable to retrieve, review articles, adult population, no fatal outcome, other types of FAOD. Reasons for exclusion marked with **: no original patient data, no fatal outcome within 14 days postpartum, unclear diagnosis of the FAOD type.

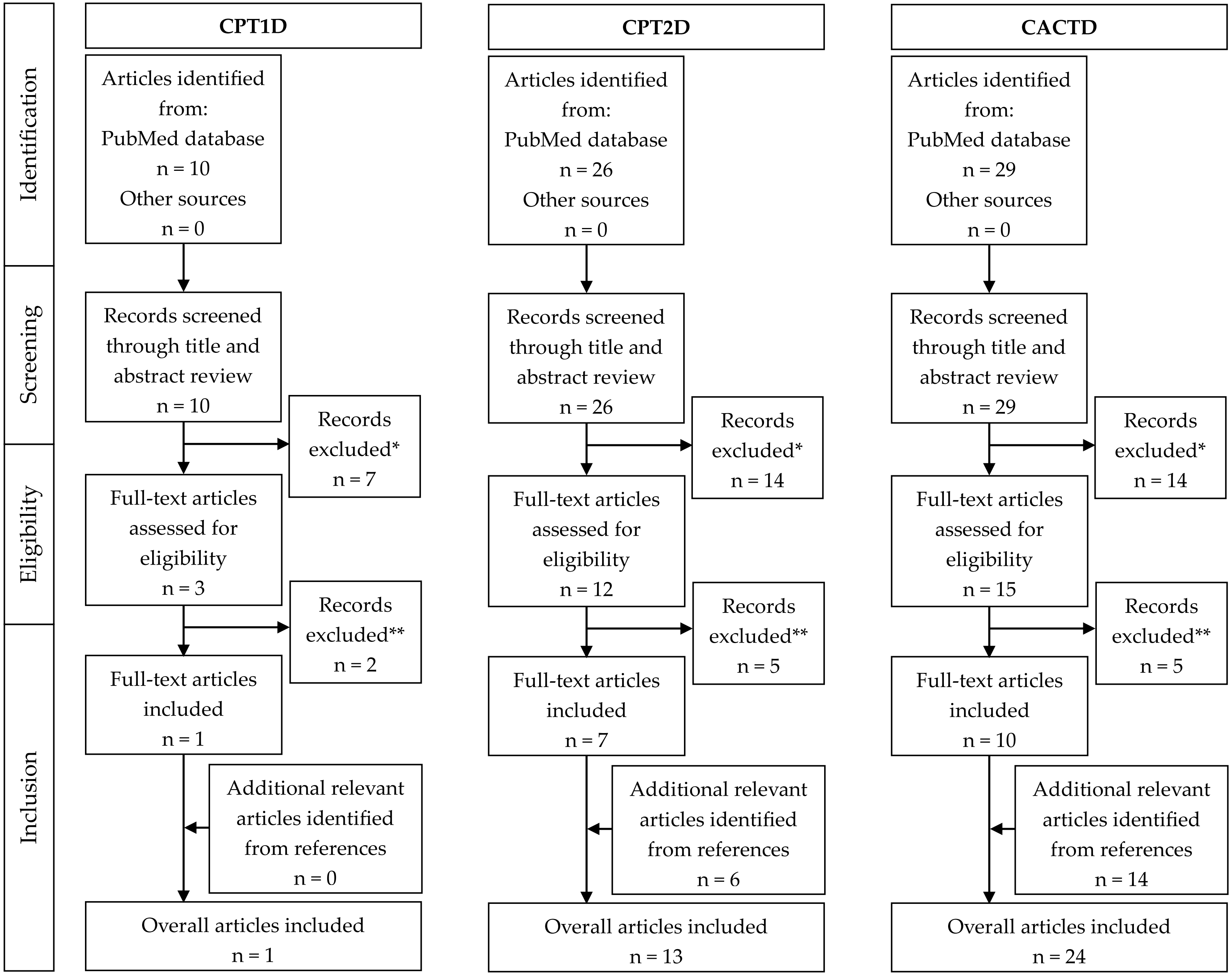


Figure S2: PRISMA flow diagram for a systematic literature review on early neonatal deaths due to FAODs. The diagram outlines a review of the following FAOD types: carnitine palmitoyltransferase I deficiency (CPT1D), carnitine palmitoyltransferase II deficiency (CPT2D), and carnitine-acylcarnitine translocase deficiency (CACT). Reasons for exclusion marked with *: no human data, articles not in English, records unable to retrieve, review articles, adult population, no fatal outcome, other types of FAOD. Reasons for exclusion marked with **: no original patient data, no fatal outcome within 14 days postpartum, unclear diagnosis of the FAOD type.
